# 1q21.1 Duplication Syndrome and Anorectal Malformations: A Literature Review and a New Case

**DOI:** 10.3390/cimb47010026

**Published:** 2025-01-03

**Authors:** Maria Minelli, Chiara Palka Bayard de Volo, Melissa Alfonsi, Serena Capanna, Elisena Morizio, Maria Enrica Miscia, Gabriele Lisi, Liborio Stuppia, Valentina Gatta

**Affiliations:** 1 Unit of Molecular Genetics, Center for Advanced Studies and Technology (CAST), University “Gabriele d’Annunzio” of Chieti-Pescara, 66100 Chieti, Italyv.gatta@unich.it (V.G.); 2 Unit of Clinical Pediatrics, “SS. Annunziata” Hospital, 66100 Chieti, Italy; chiara.palka@asl2abruzzo.it; 3 Unit of Assisted Reproductive Technologies, “Gaetano Bernabeo” Hospital, 66026 Ortona, Italy; 4 Unit of Pathological Anatomy and Histology, “San Pio da Pietrelcina” Hospital, 66054 Vasto, Italy; 5 Pediatric Surgery Unit, Department of Medicine and Aging Science, University “Gabriele d’Annunzio” of Chieti-Pescara-“Santo Spirito” Hospital, 65122 Pescara, Italygabriele.lisi@unich.it (G.L.); 6 Department of Neurosciences, Imaging and Clinical Sciences, University “Gabriele d’Annunzio” of Chieti-Pescara, 66100 Chieti, Italy

**Keywords:** ARMs, CMA, 1q21.1 duplication

## Abstract

Background: Anorectal malformations (ARMs) are a common pediatric surgical problem with an incidence of 1:1500 to 1:5000 live births. The phenotypical spectrum extends from anal stenosis to imperforate anus with or without anal fistula to persistent cloaca. They can manifest as either non-syndromic or syndromic conditions. Various environmental and genetic risk factors have been elucidated. The widespread use of genetic screening tests for the investigation of developmental disorders increased the recognition of copy number variants (CNVs) of the 1q21.1 region. Duplications have also been associated with a multitude of congenital anomalies, such as heart disease, short stature, scoliosis, urogenital, and ARMs, and they have also been found in healthy individuals. The aim of this manuscript is to contribute to the definition of the phenotype associated with 1q21.1 duplications. Case presentation: The present case describes a male, referred to us for an ARM, in whom array—comparative genomic hybridization (array-CGH) identified 1q21.1 duplication inherited from his healthy mother. No other genetic test was performed on the patient. Conclusions: We propose considering genetic evaluation and analysis in patients with only one congenital malformation in order to eventually make an early diagnosis and a better quality of treatments.

## 1. Introduction

**Figure 1:**
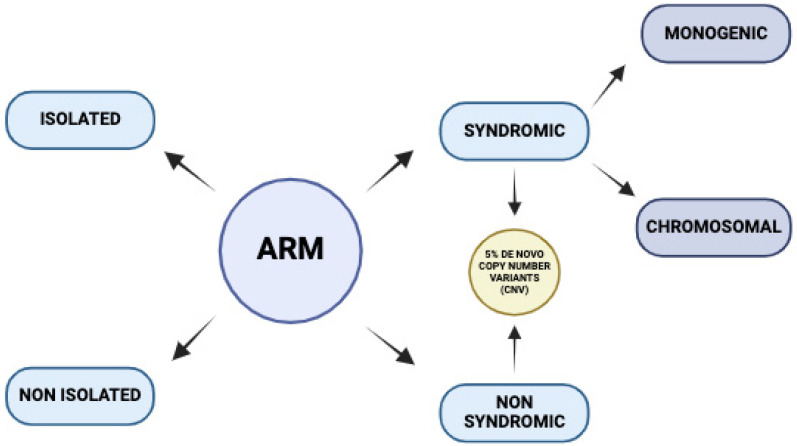
An overview of the clinical manifestations and genetic causes of ARMs.

Anorectal malformations (ARMs) are a prevalent pediatric surgical condition, with an incidence ranging from 1:1500 to 1:5000 live births, exhibiting male predominance. The Krickenbeck scoring system serves as the primary classification method for distinguishing different ARM phenotypes. The spectrum of ARM variants encompasses major clinical categories and rare regional variations. In female patients, the predominant malformations are perineal fistulas, while in males, recto-urinary fistulas are more commonly observed. [1] Examples of environmental risk factors include prenatal exposure of parents to nicotine, toxins (retinoic acid), alcohol, caffeine, and drugs during pregnancy (thalidomide, tridione, and benzodiazepines); occupational hazards; overweight/obesity and diabetes mellitus; paternal smoking; and assisted reproduction techniques. Approximately 60% of patients exhibit concomitant anomalies, with the genitourinary tract (20–54%), cardiovascular system (27%), skeletal system (43%), and gastrointestinal tract (18%) being the most commonly affected areas [2]. As illustrated in Figure 1, ARMs may present as non-syndromic (isolated, 40%) or syndromic (non-isolated, 60%) conditions.

They might appear as part of the phenotypic spectrum of many chromosomal abnormalities (up to 10% of syndromic ARM, such as Trisomy 21, Microdeletion 13q, and Tetrasomy 22pter-q11/Cat Eye syndrome) or monogenic syndromes (such as Currarino, Pallister-Hall, Townes-Brocks, Opitz G/BBB, CHARGE, Duane–Radial Ray, Walker–Warburg, Ivemark, Klippel–Feil, Kaufman–McKusick, and Lowe) [1]. Furthermore, ARMs are associated with a VATER complex in 11% of cases and VACTERL complex in 4% of cases [2]. ARMs represent a very rare feature of all of these syndromes that indeed are usually diagnosed by the presence of other typical traits. Several studies suggest that ARMs are inherited in an autosomal dominant mode [3,4,5], but the low number of patients and the incomplete penetrance of a large number of genetic condition correlated with ARMs’ etiology might present an effective possible bias together. The first report estimated the recurrence risk for siblings to be 1% [6], confirmed by other studies by Falcone R.A., Stoll C., and colleagues in 2007 [3,7]. Notably, Falcone et al. found that parents of children with perineal or vestibular fistulas had a 3% chance of having another affected family member and that parents of males or females born with perineal fistula could be told that there is a 5–7% chance of having a family member with a congenital anomaly. The recurrence risk for couples with one child affected, in the case of isolated ARMs, is estimated to be 1–2%, while in the case of VACTERL association, for example, it decreases at <1%, similar to that of the general population. The extensive implementation of genetic testing in the field of developmental disorders has enabled the identification of copy number variations (CNVs) within the 1q21.1 region. The presence of both microdeletions and microduplications in this region has been linked to a wide spectrum of pathologies, including autism spectrum disorders, attention-deficit disorder (ADHD), learning disabilities, hypotonia, facial dysmorphisms, and schizophrenia. These alterations may be either inherited or occur de novo [8]. The 1q21.1 region is regarded as being genetically unstable due to its possession of one of the largest areas of identical duplication sequences in the human genome [9], spanning approximately 1.35 Mb and encompassing at least 12 genes. This region is particularly prone to non-allelic homologous recombination (NAHR), a process in which a DNA segment is exchanged for a homologous segment, resulting in a change in the genetic composition of the cell. The delineated breakpoints (BP1-BP4) divide the 1q21.1 band into a proximal and a distal region, predisposing to two classes of CNVs. The class I is characterized by its occurrence at the 1q21.1 distal region, extending from BP3 to BP4, with a size range of 800 kb to 2 Mb. The present case report describes a male patient, referred for an ARM, in whom array—comparative genomic hybridization (array-CGH) identified a maternally inherited 1q21.1 distal duplication. The objective of this paper is to enhance the delineation of the phenotype associated with 1q21.1 duplications.

## 2. Case Presentation

The proband is a six-month-old male infant who was referred to our clinic for treatment of perineal fistula, which was treated with Y-V anuplasty. He is the second child of healthy and non-consanguineous parents, born at 40 weeks of gestation by caesarean section after a pregnancy characterized by gestational diabetes. At birth, the infant exhibited a weight of 4250 kg (98th percentile), a length of 52 cm (75th percentile), and a cephalic circumference (CC) of 38 cm (100th percentile). His Apgar score was 8 at the first minute and 9 at the fifth minute. However, at the age of two days, the infant exhibited symptoms of respiratory distress and hypocalcemia, and he was treated with oral calcium gluconate. Electrocardiography (ECG), red reflex examination, oto-acoustic emissions, renal and spinal ultrasounds, and spine X-ray results were normal, while heart ultrasounds revealed patent foramen ovale (PFO). A physical examination revealed no significant dysmorphic features; however, mild hypotonia of the upper limbs and trunk was observed. A review of the family history did not reveal any significant medical conditions. As the proband did not present with clinical features linked to a specific genetic syndrome, a chromosomal microarray (CMA) was requested, given its broad-spectrum diagnostic capacity.

**Figure 2:**
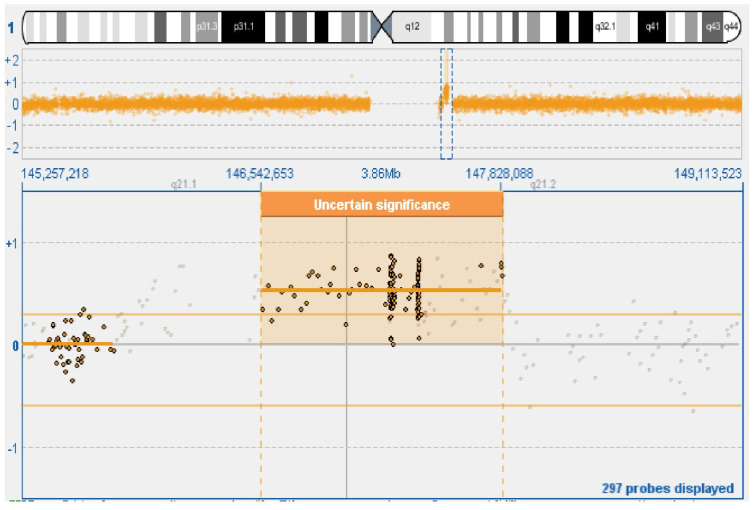
The figure shows, below the ideogram of chromosome 1, the partial duplication of the long arm in the q21.1 region detected in the proband and in his mother.

Genomic DNA of the patient and his parents was extracted from peripheral blood lymphocytes using Qiagene Amp MiniKit 40, in accordance with the manufacturers’ instructions. Proband and reference DNA were labelled with Cy5-dUTP and Cy3-dUTP, respectively. Whole-genome array-CGH was performed using CytoSure Oligo *ISCA v2 (International Standard Cytogenetic Array) 4 × 180 K with an average resolution of 100 kb (Build37: Feb 2009-hg19), in accordance with the manufacturers’ instructions. Data were analyzed with CytoSure Interpret Software 4.2.5. Array-CGH detected a 1q21.1 distal duplication in the proband that spans 1.29 Mb (146542653–147828088) and encompasses the following genes: PRKAB2, FMO5, CHD1L, BCL9, ACP6, GJA5, GJA8, and GPR89B. The rearrangement was inherited from his healthy mother (Figure 2).

## 3. Discussion

The 1q21.1 distal duplication has been associated with macrocephaly, dysmorphic features, developmental delay, intellectual disability, and autism spectrum disorders, as well as a variety of congenital anomalies without a clear genotype–phenotype correlation. On the other hand, 1q21.1 distal duplications can also be found in healthy individuals. To date, 110 individuals with 1q21.1 duplications have been reported in the literature, with highly variable inter- and intrafamilial outcomes [8,10,11,12] and only ten prenatal cases [13,14,15,16]. In a recent paper, Bourgois et al. reviewed the literature and described 34 new cases of 1q21.1 duplications. Particularly for those involving the distal region, in their cohort, almost all (92%) patients had developmental delay, 83% had psychiatric/behavioral conditions (i.e., ADHD, autism spectrum disorder, and mood disorders), 67% had ocular anomalies (i.e., strabismus, nystagmus, visual impairment, and cataract), three out of four had cardiovascular anomalies, 50% had macrocephaly, and all had at least one facial dysmorphism. Rarely, they may present with gastrointestinal, genitourinary, musculoskeletal, and growth parameter abnormalities [17]. In the literature, neurodevelopmental disorders were reported in 60% of patients, 32% had psychiatric/behavioral conditions, macrocephaly was reported in 36% of patients, and 43% of these had a dysmorphic feature; of those who underwent cardiac and cerebral examination, 64% had a cardiovascular abnormality and 40% had brain abnormalities. The incomplete penetrance and variable expressivity of this condition pose challenges for counselling regarding prenatal diagnosis, especially for the association with a possible and variable neurodevelopmental phenotype that cannot be predicted prenatally [16]. On the other hand, the vast majority of ARM cases are first recognized at birth, as prenatal detection is still rare, with a detection rate of only 16%.

More recently, in 2021, Marcelis et al. [18] proposed a guideline to study patients with ARM, first looking at the clinical history, which includes the following:

- The mode of conception, since it is known that assisted reproduction can increase the risk of ARMs;
- Maternal illness during pregnancy (especially diabetes);
- The potential use of medication;
- Prenatal investigations (ultrasound and genetic), given that oligohydramnios can be indicative of renal or genitourinary anomalies, while polyhydramnios might be an indication of esophageal atresia in VACTERL patients.

**Figure 3:**
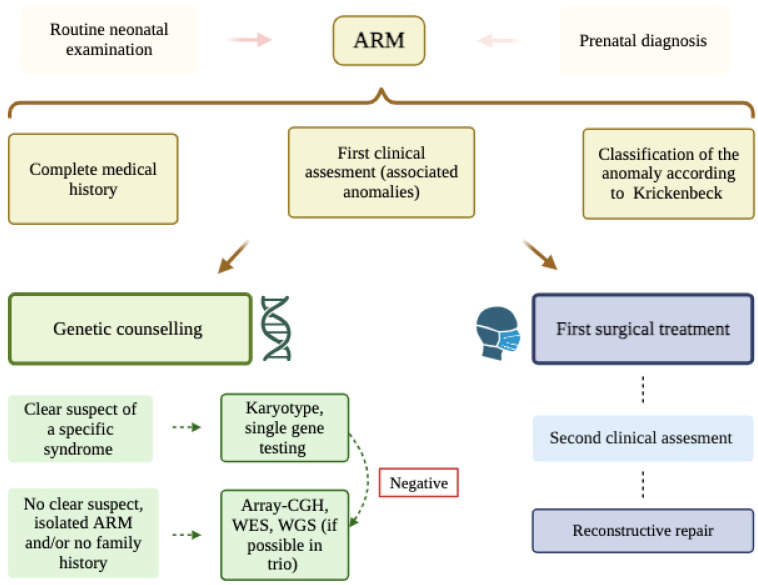
Algorithm for the clinical evaluation of a patient diagnosed with ARM (created in BioRender. Minelli, M. (2024); https://BioRender.com/h05w499, accessed on 14 November 2024).

It is important to review the family history with a pedigree of at least three generations, looking for other family members with ARM or a history of constipation (which could be a sign of unrecognized ARM), other congenital anomalies (digital changes, external ear abnormalities, and hearing loss), developmental delay or intellectual disability, and a history of stillbirth, which could indicate a balanced chromosomal aberration (translocation/inversion) in the family. If ARM is suspected after birth, the initial clinical evaluation should be aimed at classifying the type of ARM (Krickenbeck) and identifying other major anomalies that may affect the prognosis and choice of treatment before the first operation. After the initial surgical treatment, a second clinical evaluation should be performed (dysmorphology, imaging of the spinal cord and spine, limb and genital anomalies, and hearing loss) to look for signs of specific syndromes. In any case, long-term follow-up of the patient is also necessary; in fact, in many syndromic forms of ARM, symptoms such as developmental delay or mental retardation only become apparent later in life, and their early detection is important in order to initiate appropriate preventive and therapeutic interventions. Currently, there is no specific place for routine genetic testing in patients with isolated ARM and no suspicious family history. If there is a clear suspect of a specific syndrome, karyotype or single-gene testing is recommended. If there is not a clear suspect, if first-tier analysis results are negative, or if the results partially explain the phenotype, then broad analysis, like microarray or WES (Whole Exome Sequencing), should be performed, possibly in trios and after the careful genetic counselling of parents because of the risk of incidental findings of conditions divergent from ARM [19] (Figure 3). Many laboratories also use WGS (Whole Genome Sequencing) for conducting analysis in trios in order to find submicroscopic deletions or duplications. WES and WGS can be very useful in the case of single nucleotide variants in genes that can act as a modifier, a deep intronic variant, or structural abnormalities that cannot be analyzed using array-CGH. On the other hand, the use of WGS and WES as first- or second-tier and in singleton or in trio pregnancies depends on the specific clinical center and its economic potential.

**Figure 4:**
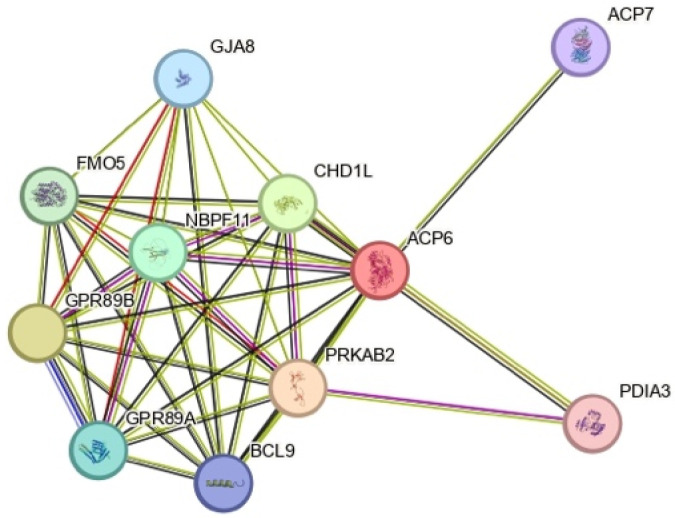
“STRING” reproduction of the ACP6 pattern of interactions.

In this report, we describe a young male referred to us for a surgically treated perineal fistula, in whom a 1q21.1 duplication was identified using array-CGH, inherited from his mother. In particular, ARMs are a very rare feature of this condition. According to the current literature, there are only two other patients with both ARMs and 1q21.1 duplications detected by array-CGH analysis: a girl with retro-perineal fistula and a 1.3 Mb duplication disrupting the ACP6 gene [1], and a 3-year-old male with imperforate anus and perineal fistula and a 1.3 Mb duplication (146,508,774–147,825,454) [19]. Both subjects had no additional features. Of all the genes included in the distal region duplicated, GJA5 has been associated with autosomal dominant atrial fibrillation, tetralogy of Fallot, and other cardiac defects so it can contribute to the cardiac phenotype [17,20]; on the other hand, GJA8 has been correlated to autosomal dominant cataract and other ocular anomalies. Regarding the ARMs, the BCL9 gene, already proposed as a risk gene for neuropsychiatric disorders [21], can be seen as a strong candidate due to its expression in intestinal tissues and its involvement in the WNT signaling pathway [22], which is known to be one of the pathways hypothesized to be causative in the etiology of ARMs, but further studies are also needed considering that its overexpression and possible epigenetic signature could affect its functions. In Figure 4, starting from ACP6, it is clear that each individual gene of interest in the critical region of the 1q21.1 duplication is linked in co-expression with the others, but meaningful experimental studies are needed to establish this correlation and their specific role in the 1q21.1 duplication phenotype.

The occurrence of the same duplication in our patient’s healthy mother could be explained by the incomplete penetrance of phenotypic expression that characterizes this genetic condition. Unfortunately, it was not possible to test any other relatives and to follow the clinical course of our proband because the family lived in another region. Therefore, we cannot exclude the possibility of developing a neuropsychiatric or behavioral disorder. In any case, our results extend the clinical picture with the finding of another patient with anorectal manifestation. We also propose considering genetic evaluation and analysis in patients with only one congenital malformation in order to allow an early diagnosis and a better quality of treatment. Studies are also needed to better define the 1q21.1 duplication syndrome and to understand how the overexpression of genes in this region can cause this complex phenotype.

## Data Availability

The datasets used and/or analyzed during the current study are available from the corresponding author on reasonable request.
